# High-Affinity Detection of Alpha-Synuclein by Aptamer-Gold Conjugates on an Amine-Modified Dielectric Surface

**DOI:** 10.1155/2019/6526850

**Published:** 2019-11-30

**Authors:** Xuemei You, Subash C. B. Gopinath, Thangavel Lakshmipriya, Dingan Li

**Affiliations:** ^1^Department of Neurology, The Fourth People's Hospital of Shaanxi Province, No. 512 Xianning East Road, Xincheng District, Xi'an, Shaanxi 710043, China; ^2^School of Bioprocess Engineering, Universiti Malaysia Perlis, 02600 Arau, Perlis, Malaysia; ^3^Institute of Nano Electronic Engineering, Universiti Malaysia Perlis, 01000 Kangar, Perlis, Malaysia; ^4^Department of Neurology, Hanzhong Central Hospital, 22 Kangfu Road, Hanzhong, Shaanxi 723000, China

## Abstract

Parkinson's disease (PD) is a progressive health issue and influences an increasingly larger number of people, especially at older ages, affecting the central nervous system (CNS). Alpha-synuclein is a biomarker closely correlated with the CNS and PD. The loss of neuronal cells in the substantia nigra leads to the aggregation of alpha-synuclein in the form of Lewy bodies, and Lewy neuritis is a neuropathological hallmark. The therapeutic approach of PD focuses on alpha-synuclein as an important substrate of PD pathology. So far, research has focused on antialpha*-*synuclein to minimize the burden of extracellular alpha-synuclein in the brain, and as a consequence, it ameliorates inflammation. Interdigitated electrode (IDE) biosensors are efficient tools for detecting various analytes and were chosen in this study to detect alpha-synuclein on amine-modified surfaces by using antiaptamer-alpha-synuclein as the probe. In addition, a gold nanoparticle-conjugated aptamer was used to enhance the detection limit. The limit of detection for the binding between alpha-synuclein and aptamer was found to be 10 pM. Control experiments were performed with two closely related proteins, amyloid-beta and tau, to reveal the specificity; the results show that the aptamer only recognized alpha-synuclein. The proposed strategy helps to identify the binding of aptamer and alpha-synuclein and provides a possible method to lower alpha-synuclein levels and inflammation in PD patients.

## 1. Introduction

Neurogenerative disorders lead to neuron complications, and the causative illnesses are Parkinson's, Alzheimer's, and Huntington's diseases. Parkinson's disease (PD) affects the central nervous system by acting primarily on the cells in the substantia nigra [1, 2]. Issues in the neurons cause impaired routine movement, speech problems, difficulty in balancing, muscle rigidity, and tremors [3, 4]. Alpha-synuclein is a presynaptic neuronal protein linked genetically and neuropathologically to PD, and it contributes to pathogenesis in different ways [5, 6]. Generally, it has been thought that an aberrant soluble oligomeric conformation of alpha-synuclein, called “protofibril,” could mediate the disruption of cellular homeostasis, causing neuronal death. In particular, a protein aggregation process, called seeded aggregation, produces the protofibril, which can reach neighboring cells through a mechanism known as transcellular propagation [7, 8]. The proper diagnosis of PD is mandatory to provide patients with appropriate treatment and to improve the quality of their everyday life [1, 9]. A therapeutic approach using alpha-synuclein has been demonstrated due to its association with PD pathology. The primary aim of this type of therapy is to minimize the extracellular amount of alpha-synuclein in the brain to reduce neuroinflammation. Currently, passive immunization through the administration of antibodies generated against alpha-synuclein is one of the most promising ways to achieve this potential goal [10]. In this work, an aptamer immobilized on the surface of an interdigitated electrode (IDE) was used as a probe capable of binding alpha-synuclein. IDE, a dielectric biosensor, has been used for the detection of several targets at low concentrations [11, 12]. This kind of chemical sensor has many advantages, such as easy setup, low detection limit, high robustness, and low sample volume consumption. In IDEs, biological events are converted into an electrical signal. In the current study, this signal is produced by the binding between the aptamer and alpha-synuclein.

An aptamer is an “artificial antibody” selected from a randomized group of nucleic acids that involves three simple steps: binding, separation, and amplification [13, 14]. This *in vitro* selection technique is called “SELEX” and allows, from a pool of DNA or RNA, production of a wide range of “artificial antibodies” capable of specifically binding a target. To obtain this prepared nucleic acid pool, complexation is performed under optimal conditions, and the nucleic acids bound to the target are separated by any separation method. The nucleic acids attached to the target are amplified, usually by polymerase chain reaction, and the steps are repeated multiple times. After approximately 10 cycles, the nucleic acid with high affinity to the target is identified by sequencing. Since aptamers have more advantages than “natural antibodies,” the application of aptamers is widely welcomed in all types of fields, such as environmental, medical, drug delivery, and imaging [13, 15, 16]. Among other detection probes, aptamers have more been specifically used in the diagnosing field due to their high sensitivity and specificity to the target [17, 18]. Aptamers have more sensitive and specific binding ability because they require fewer bases to match with the partner molecule. This feature facilitates the discrimination of targets from closely related molecules by the aptamer. In addition, if the capturing molecule (aptamer) is more sensitive to the target, then the aptamer is automatically prone to detect smaller amounts of the desired target [19]. Further, to enhance the detection system, we conjugated aptamers with gold nanoparticles (GNPs) and alpha-synuclein was detected.

Nanoparticles are widely used in the field of biosensors due to their low detection limit [20, 21]. Among other metal/nonmetal particles, GNPs show excellent thermal and optical properties and have good binding stability with biomolecules [22, 23]. Conjugation of a probe or analyte with GNPs has been shown to improve the detection system, leading to high performance in target validation [24, 25]. To utilize these advantages, in this work, we combined aptamers with GNPs to create capture probes capable of detecting alpha-synuclein. The strategy demonstrated in this research aims to use aptamers to minimize alpha-synuclein levels and, consequently, the severity of inflammation.

## 2. Materials and Methods

### 2.1. Reagents and Biomolecules

Alpha-synuclein, (3-aminopropyl)triethoxysilane (APTES), bovine serum albumin (BSA), ethanolamine, gold nanoparticles (30 nm), sodium hydroxide (NaOH), and phosphate-buffered saline (PBS) were purchased from Sigma-Aldrich (USA). The antisynuclein aptamer (5′-ATAGTCCCATCATTCATTGTATGGTACGGCGCGGTGGCGGGTGCGTGGAGATATTAGCAAGTGTCA-3′) was synthesized commercially from a local supplier. All other reagents used were analytical grade and stored as recommended.

### 2.2. Interdigitated Electrode (IDE) Sensing Surface Preparation

The silver IDE electrode was printed on a (100) silicon wafer sample using the traditional wet-etching method. Positive photoresist (PR) was deposited by spin coating on the silicon wafer, followed by a soft bake for 90 sec. Ultraviolet (UV) light exposure was conducted for 10 sec to allow pattern transfer from the IDE mask onto the surface of the sample. Next, the development process was carried out for 15 sec by using an RD-6 developer. Then, the sample was baked at 110°C to remove unwanted moisture and improve the adhesion between the silver and SiO_2_ layer. Finally, the unexposed area was removed using a silver etchant for 23 sec and then cleaned with acetone. Using this basic modification, further development was performed using chemical functionalization. The success of this fabrication was confirmed by high-resolution microscopy techniques (3D nanoprofiler and high-power microscopy), as shown in our earlier studies [11, 12].

### 2.3. Preparation of Aptamer-GNP Conjugates

To find the suitable concentration of aptamer to cover the entire surface of the GNPs, different concentrations of aptamer (final concentrations were 1–3 *µ*M) were mixed independently with a constant volume (10 *µ*l) of GNPs and kept at room temperature for 30 min. Next, bound GNP-aptamer conjugates were separated by centrifugation (10,000 ×g for 10 min). Then, the samples were washed thoroughly five times with distilled water and separated by another centrifugation with the same parameters. Finally, the obtained aptamer-GNP conjugate as a precipitate was kept at 4°C and used for further experiments.

### 2.4. Stability Assay of Aptamer-GNP Conjugates

The stability of the aptamer-GNP conjugates was analyzed by salt-induced aggregation. For that process, an NaCl solution at high concentration (800 mM) was added to 1, 2, and 3 *μ*M solutions of the prepared aptamer-conjugated GNPs. The solution was left to settle for 10 min at RT to observe the aggregation of the GNPs in the presence/absence of aptamers. The samples were then analyzed with a UV-Vis spectrophotometer to observe the variations in the absorption spectrum, which were determined in the wavelength range of 400–700 nm.

### 2.5. GNP-Aptamer Attachment on an Amine-Modified IDE Surface

After confirming the suitable concentration of aptamer to be immobilized on the GNP surface, the prepared aptamer-GNP probe was immobilized on the surface of an amine-modified IDE. For that process, first, 2% APTES (diluted in 30% ethanol) was dropped on the IDE surface and maintained for 4 h at RT; then, the surface was washed with 30% ethanol five times. Next, 10 *µ*L of prepared aptamer-GNP conjugates was added to the surface and maintained for 30 min at RT to allow the chemical interaction of GNP with the amine.

### 2.6. Detection of Alpha-Synuclein on the GNP-Aptamer-Modified Surface

The aptamer-GNP probe immobilized on the IDE surface was used to detect alpha-synuclein. For that process, after the probe was immobilized, the remaining sensing surface was blocked by adding 10 *µ*L of 1 M ethanolamine. This reaction took place for 30 min at RT. To detect alpha-synuclein, 1 *µ*M alpha-synuclein was dropped on the aptamer-GNP-immobilized IDE surface. The binding between aptamer and alpha-synuclein was detected by monitoring the change in current levels. For each immobilization, the current level was recorded and then compared with the other values.

### 2.7. High-Performance Analytical Study: Sensitivity and Specificity

To evaluate the detection limit of the sensor, different alpha-synuclein concentrations were independently titrated from low to high (10 pM to 1 *μ*M) and added to the aptamer-GNP-modified IDE surface. The current changes indicated the binding of alpha-synuclein with its aptamer. The current changes were plotted to find the limit of detection.

The specific detection of alpha-synuclein was carried out with two closely related proteins, amyloid-beta and tau. These two proteins were allowed to interact with the aptamer-GNP conjugate probe immobilized on the IDE sensing surface after the surface was covered with ethanolamine. The current changes were recorded and plotted independently.

## 3. Results and Discussion

**Figure 1:**
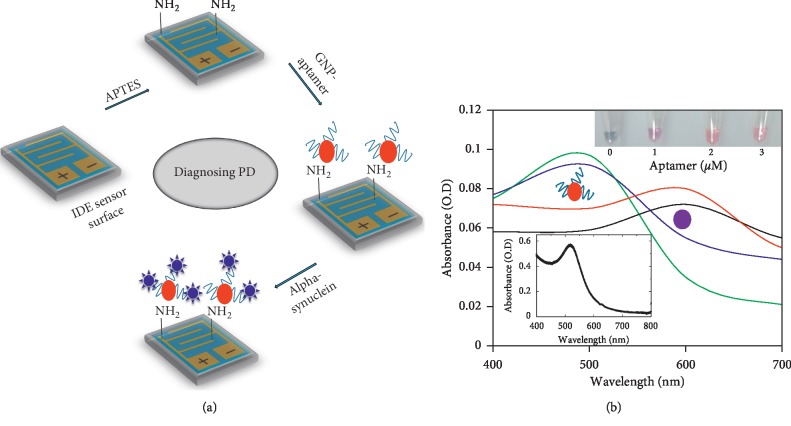
(a) Schematic representation of alpha-synuclein detection by an aptamer-gold nanoparticle probe on an IDE surface. The surface was modified by APTES to immobilize the GNP-aptamer, and then, alpha-synuclein was detected. (b) Colorimetric analysis to reveal the stability of aptamer attachment on the GNPs. Analyses were performed by both colorimetric (figure inset) and spectrophotometric methods. The figure inset graph represents the absorbance maximum of as-received GNPs.

PD is a neurodegenerative disorder that significantly affects the daily activity of elderly people. Genetics play a crucial role in causing PD [26]. Appropriate clinical treatment is necessary to treat PD and improve quality of life [17]. Various physical activities have been recommended to help the recovery of PD patients. However, to inform proper treatment, a suitable probe-based biomarker analysis is necessary [27]. In this research, the aptamer sequence was adapted as the probe to detect alpha-synuclein at a low level on an amine-modified IDE sensing surface [28, 29].  Figure 1(a) shows a schematic representation for detecting alpha-synuclein by an aptamer-based probe. As shown in the figure, the first step is the immobilization of the aptamer-GNP conjugates on the surface of an IDE previously functionalized with APTES and then the reaction with alpha-synuclein.

### 3.1. Stability Assay of Aptamer-GNP Conjugates

Considering that the aptamer-GNP complex was used as a probe in the present work, the concentration of the aptamer necessary to cover the entire surface of a GNP was analyzed. For this optimization, different concentrations of aptamers were added independently to the GNPs, and 800 mM NaCl was added to these conjugates to check the stability. As shown in Figure 1(b), in the absence of aptamers, the GNPs aggregate due to NaCl and the color of the solution turns blue. UV-Vis spectroscopy detected the maximum absorption peak in the scanned region from 400 to 700 nm. When a 1 *μ*M aptamer solution is added to the GNPs, the solution turns pink. This effect is due to the conjugation of aptamer with GNPs, which reduces the nanoparticle aggregation. The color intensity of the solution increases with an increasing concentration of aptamers. The original red color (dispersion) was retained at higher concentrations of the aptamer, and the UV results confirm its peak maximum at ∼520 nm (Figure 1(b)).

### 3.2. Preparation of IDE Sensing Surface

**Figure 2:**
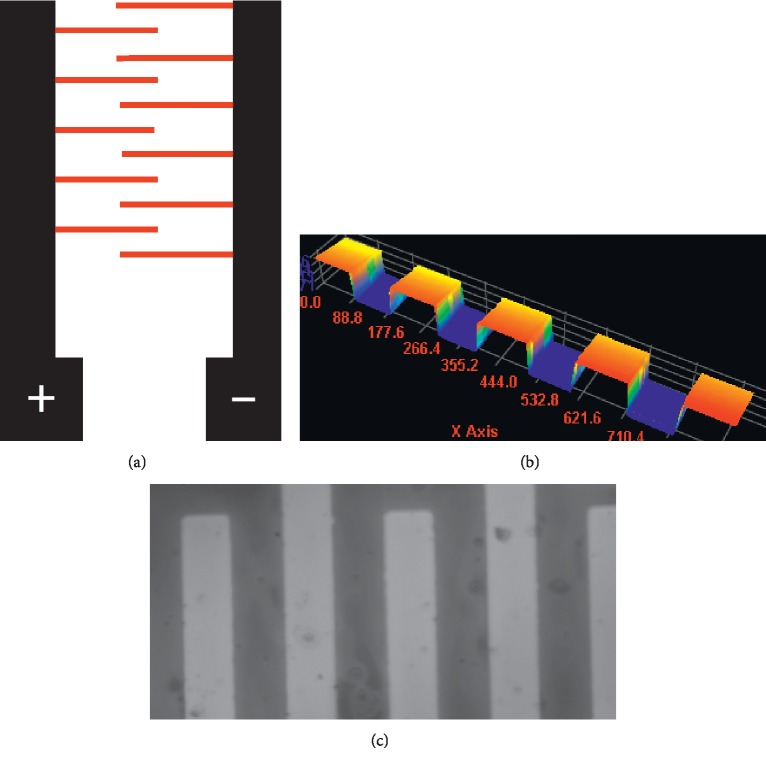
Surface analysis of the IDE. (a) Diagram representation of the dielectric sensor. Both positive and negative electrodes are shown with the finger regions. (b) The surface of the IDE sensor gap regions is displayed by a 3D nanoprofiler. (c) The surface of IDE sensor gap regions is displayed by high-power microscopy.

For the detection of alpha-synuclein, the fabrication of the IDE surface was performed by a standard lithography technique as optimized in our previous work [11, 12]. The gap and finger pattern of IDE was created on silica and aluminum substrate materials and used for biomolecular analysis (Figure 2(a)). To characterize the surface of the IDE, morphological analyses were carried out by 3D nanoprofiling and high-power microscopy. The images obtained from these high-resolution analyses show the uniformity of the pattern and the absence of surface distortions (Figures 2(b) and 2(c)). This optimal surface was used to evaluate the ability of the probe (aptamer-GNP conjugates), which is attached to the IDE sensing amine-modified surface, to detect alpha-synuclein.

### 3.3. Detecting Alpha-Synuclein on Surfaces with an Immobilized Aptamer-GNP Probe

**Figure 3:**
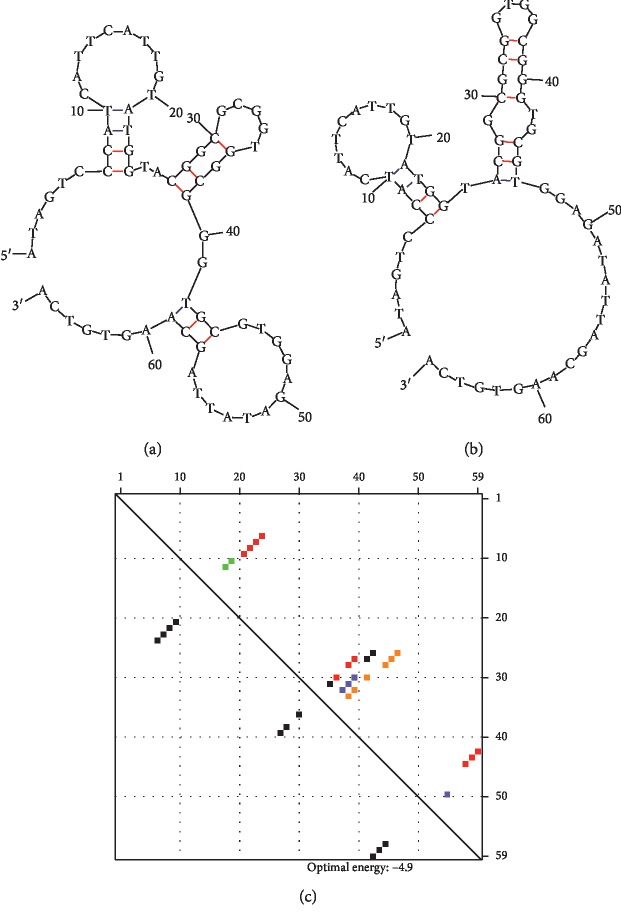
Possible secondary structures of the alpha-synuclein aptamer. (a) Structure 1. (b) Structure 2. Both structures show 4 loop and 3 stem regions. (c) Comparison and energy levels (dG) of both structures.

**Figure 4:**
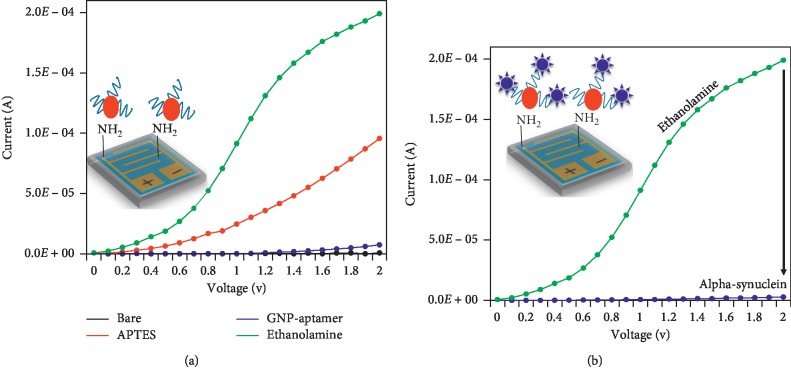
(a) GNP-aptamer immobilization on the IDE sensing surface. The IDE surface was modified by APTES. The GNP-aptamer was immobilized, and the remaining surfaces were blocked by ethanolamine. (b) Alpha-synuclein detection on a GNP-aptamer-modified IDE sensing surface. A schematic representation is shown in the figure inset.

The folding pattern of the aptamer was analyzed by mfold-online software, and the output displayed the possibility of two different structures. Both structures appear to have 4 loops and 3 stems that are different and are correctly folded (Figures 3(a) and 3(b)); however, the energy levels (dG) of these structures are similar (Figure 3(c)).  Figure 4(a) explains the current changes due to the biomolecules after each immobilization step on the IDE surface. The bare surface of the IDE (black) showed a current of 7.65*E*^−07^, and after attaching APTES, the current increased to 9.56*E*^−05^. When the aptamer-GNP conjugates were added, the current decreased to 7.43*E*^−06^, which might be due to the charge differences of the GNP-aptamer conjugates. These current changes clearly confirmed the binding of the aptamer-GNP probe on the IDE surface. The uncoated surface was blocked with ethanolamine, producing a drastic change in the current from 7.43*E*^−06^ to 1.99*E*^−04^. These current changes are due to the presence of ethanolamine on a large amount of the free surface area of the IDE and on the surface of the GNPs. This coverage helps to avoid the nonspecific binding of alpha-synuclein on the sensing surfaces and the GNP surfaces. After these preparation steps, 1 *µ*M alpha-synuclein was added, and a clear change in the current was observed (Figure 4(b)). Upon adding alpha-synuclein, the current was decreased to 2.55*E*^−06^. This result confirms the binding of alpha-synuclein with the aptamer. Moreover, due to the GNP-aptamer conjugates, there was a larger amount of aptamer bound on the surface of the IDE, so a higher difference in current was observed.

### 3.4. Limit of Detection of Alpha-Synuclein

**Figure 5:**
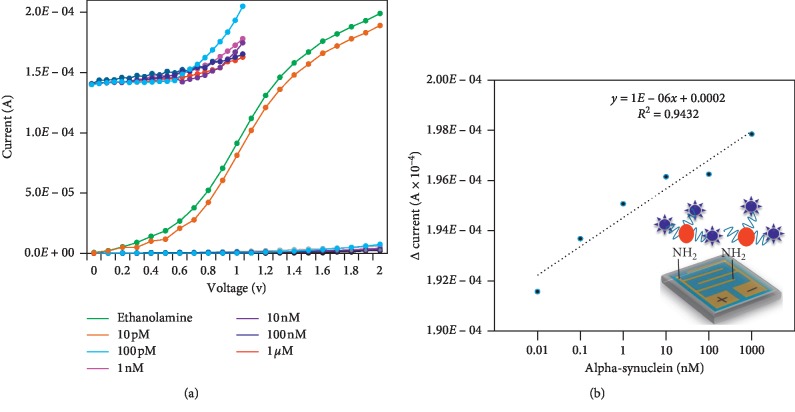
(a) Dose-dependent binding of alpha-synuclein on the GNP-aptamer-modified IDE sensing surface. Alpha-synuclein concentrations from 0.1 to 1000 pM were tested independently on the IDE surface. (b) Linear regression analysis with different concentrations of alpha-synuclein. The limit of detection was 10 pM. Schematic representations are shown in the figure insets.

Since 1 *µ*M alpha-synuclein was clearly detected on the aptamer-GNP-modified surface, to determine the limit of detection, different concentrations of alpha-synuclein (10 pM to 1 *μ*M) were independently tested on the modified surface of the probe. When 10 pM alpha-synuclein was added, a slight change in current was observed. At the same time, with increasing alpha-synuclein concentration, the current decreased accordingly (Figure 5(a) and inset). At a concentration of 10 pM, the current level was 2.09*E*^−04^. The differences between the ethanolamine-attached surface and 10 pM alpha-synuclein binding with the aptamer were clearly observed. After adding 100 pM alpha-synuclein, the current decreased to 2.55*E*^−06^. Then, a prominent decrease in the current in all the concentrations of alpha-synuclein was clearly observed. From these results, it was concluded that the sensitivity limit was 10 pM alpha-synuclein by using aptamer-GNP as the probe.  Figure 5(b) displays the linear regression analysis with different concentrations of alpha-synuclein and a constant level of aptamer. The coefficient of determination obtained from the regression analysis indicates that the obtained value is reliable as it is close to “one.” Using this curve, a 3*σ* calculation was made. It is clear that the limit of detection is 10 pM.

### 3.5. High-Performance Analysis: Specific Detection of Alpha-Synuclein

**Figure 6:**
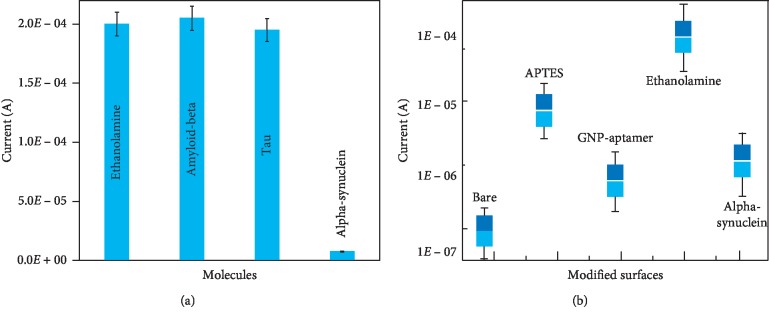
High-performance analysis. (a) Specificity analysis using two closely related proteins, amyloid-beta and tau. The figure shows that the antialpha-synuclein aptamer specifically recognizes alpha-synuclein, while the other aptamers do not show apparent current changes. (b) Reproducibility of different surface modifications on different devices.

Specific detection of alpha-synuclein was carried out on the aptamer-GNP-modified IDE surfaces. For this analysis, two closely related proteins, namely, amyloid-beta and tau, on the same aptamer-GNP-modified surfaces were independently tested. As shown in Figure 6(a), only alpha-synuclein caused a current change, while the other control proteins did not show any significant changes in current from the baseline level of the ethanolamine-blocked surface. This result highlights that alpha-synuclein was specifically detected on the aptamer-GNP-modified IDE surface without any fouling effects. The selectivity analysis was also similarly carried out with different surface modifications.  Figure 6(b) shows that there is no significant difference for any of the above molecular attachment/binding steps after repeated testing, demonstrating that the dielectric sensor exhibits high performance.

## 4. Conclusions

Parkinson's disease (PD) is a neurogenerative disorder that mainly affects older people. Diagnosing PD with a suitable biomarker helps to treat the disease and allows patients to live a healthier life. In this work, we analyzed the binding of an aptamer probe to detect alpha-synuclein. Gold nanoparticle-conjugated aptamers were attached on an interdigitated dielectric surface for analysis of the target alpha-synuclein. The limit of detection was found to be 10 pM, with high performance. The current method is viable and promising for use in detecting a wide range of biomarkers.

## Data Availability

All the data are fully available without restriction.

## Abbreviations

PD: Parkinson's disease
CNS: Central nervous system
nM: Nanomolar
nm: Nanometer
mM: Millimolar
*µ*l: Microliter
SELEX: Systematic evaluation of ligands by exponential enrichment
GNP: Gold nanoparticle
IDE: Interdigitated electrode
APTES: (3-Aminopropyl)triethoxysilane
BSA: Bovine serum albumin
NaOH: Sodium hydroxide
PBS: Phosphate-buffered saline
PR: Positive photoresist
UV: Ultraviolet.
